# Correlation of Serum CA-125 and Progesterone Levels with Ultrasound Markers in The Prediction of Pregnancy Outcome in Threatened Miscarriage

**DOI:** 10.22074/ijfs.2015.4609

**Published:** 2015-12-23

**Authors:** Maged Al Mohamady, Ghada Abdel Fattah, Eman Elkattan, Rasha Bayoumy, Dalia Ahmed Hamed

**Affiliations:** 1 Departement of Obstetrics and Gynecology, Cairo University, Giza, Egypt; 2 Department of Chemical Pathology, Cairo University, Giza, Egypt

**Keywords:** First Trimester, Ultrasound, CA-125, Progesterone, Threatened Miscarriage

## Abstract

**Background:**

The aim of this study was to evaluate the relationship between ultrasonographic findings and serum progesterone and cancer antigen-125 (CA-125) levels in threatened miscarriage and to predict pregnancy outcome.

**Materials and Methods:**

In a prospective comparative case-control study, serum CA-125 and progesterone levels were measured for 100 pregnant women with threatened miscarriage who attended the outpatient clinic or the causality department of Obstetrics and Gynecology at Kasr El-Aini Hospital, Giza, Egypt, during the period from March 2013 to October 2013. Ultrasound was performed for fetal viability, crown-rump length (CRL), gestational sac diameter (GSD) and fetal heart rate (FHR). The patients were followed up and divided into two groups based on the outcome: 20 women who miscarried (group 1), and 80 women who continued pregnancy (group 2). The sensitivity, specificity, positive predictive value (PPV), negative predictive value (NPV), and overall accuracy were tested for CA-125 and progesterone levels in prediction of the pregnancy outcome. Correlation of these chemical markers with the ultrasound markers was also examined.

**Results:**

In the group that miscarried, CA-125 level was significantly higher (P<0.001) and serum progesterone level was significantly lower (P<0.001). For prediction of the outcome of pregnancy, the cut-off limit of 31.2 IU/ml for CA-125 level yielded sensitivity, specificity and an overall accuracy of 96.2, 100 and 99.4% respectively. The cut-off limit of 11.5 ng/ml for progesterone level yielded sensitivity, specificity and an overall accuracy of 97.5, 100 and 99.8% respectively. CA-125 level had a negative correlation with progesterone level and FHR levels (r=-0.716, P<0.001) and (r=-0.414, P<0.001) respectively. Serum progesterone level correlated with GSD (r=0.521, P<0.001) and with CRL (r=0.407, P<0.001) and FHR (r=0.363, P<0.001). CA-125 level was significantly higher in the group that showed hematoma as compared with the group without hematoma (P<0.001). Also, serum progesterone level was significantly lower in the group that showed hematoma as compared with the group without hematoma (P=0.017).

**Conclusion:**

Serum CA-125 and progesterone levels are valid early predictors of the outcome of pregnancy in women with threatened miscarriage. They are correlated with some ultrasonographic markers (GSD, CRL, and FHR).

## Introduction

First-trimester bleeding is one of the most common obstetric complications, occurring in 25% of all pregnancies (1). More than 80 percent of abortions occur in the first 12 weeks of pregnancy and at least half result from chromosomal anomalies. After the first trimester, both the abortion rate and the incidence of chromosomal anomalies decrease (2). The clinical diagnosis of threatened miscarriage is presumed when bloody vaginal discharge or bleeding appears through a closed cervical os during the first half of pregnancy (3). Ultrasonography, serial serum quantitative assessment of B-subunit of human chorionic gonadotropins (B-hCG), serum cancer antigen -125 (CA-125) and serum progesterone values measured alone or in various combinations, have proven helpful in ascertaining if a live intrauterine pregnancy is present (2). Maternal serum biochemistry has also been proposed as a predictor. La Marca et al. (4) reported that the presence of low concentrations of hCG in women with threatened abortion suggests a negative outcome for the pregnancy. Progesterone concentrations show a narrow variation in the first trimester. The lowest serum progesterone concentration associated with a viable first trimester pregnancy is 5.1 ng/ml and a single serum progesterone measurement of at least 25 ng/ml carries a 97% likelihood for viable intrauterine pregnancy, being more sensitive than two serial hCG measurements (5).

In this study we examined the diagnostic accuracy of serum CA-125 and progesterone levels in the prediction of the outcome of pregnancy in patients with threatened miscarriage, as well as the correlation between these chemical markers and ultrasound markers.

## Materials and Methods

### Patients

prospective comparative case-control study was set up to determine the accuracy of biochemical markers in the prediction of the pregnancy outcome, as well as their correlation with each other and with the ultrasound markers of pregnancy outcome. The hospital ethical committee approval was attained before beginning the study. A total of 105 patients with clinical diagnosis of first trimester miscarriage were recruited in this study. They attended the outpatient clinic or the causality department of Obstetrics and Gynecology, in Kasr El-Aini Hospital, Giza, Egypt during the period from March 2013 to October 2013. Ethical committee approval of the Department of Obstetrics and Gynecology of Cairo University was obtained was obtained. All participants gave an informed consent and had preoperative clinical evaluation. Five patients did not complete their follow up program with us, and hence were considered as drop out cases leaving 100 patients who were eligible for analysis.

We included the patients that were diagnosed by 1st trimester threatened miscarriage. The patients had a singleton spontaneous pregnancy and were presenting with vaginal bleeding or spotting. The pregnancy was confirmed by a visible gestational sac of a living embryo, verified by cardiac activity visualized on real time ultrasound. The maternal age should range between 20-40 years and the gestational age should range between 7-13 weeks (calculated from the 1st day of the last normal menstrual period, preceded by 3 regular menstrual cycles, and correlating with ultrasound measurements).

We excluded patients with history of general medical disease e.g. diabetes or thyroid disease, presence of local (gynecological) disease e.g. fibroid or adnexal masses verified by normal appearance of the uterus and ovaries by ultrasound, presence of uterine malformations e.g hypoplastic uterus or septate uterus. Patients with history of recurrent miscarriages were excluded from the study; also we excluded patients with history of any maternal disease that would cause an increase in CA-125 level such as chronic pelvic infection and endometriosis. We excluded abnormal findings in the dating scan as blighted ovum or missed miscarriage. All the patients underwent vaginal examination to assess if there is any cervical dilatation as well as the amount of bleeding. They all underwent ultrasonographic and biochemical studies.

### Ultrasound studies

Each participant in the study underwent preliminary ultrasound examination. The same experienced operator performed the ultrasound examinations. Ultrasound scanning was performed to all patients using Accuvix (Medison, Korea) scanner 4-7 MHz endovaginal probe. The scan was done to assess the gestational age and fetal viability as well as to exclude any uterine malformations.

Ultrasound parameters also included gestational sac diameter (GSD), fetal crown-rump length (CRL diameter), fetal heart rate (FHR) and the presence/absence of sub-chorionic hematoma (collection between the uterine wall and the chorionic membrane).

### Biochemical studies

All venous samples (5 ml) were allowed to clot, and sera were separated by centrifugation at room temperature at 3,000 rpm for 10 minutes. Sera were stored at -80˚C until they were analyzed at the end of the study. Quantification of CA-125, and progesterone was performed using the direct chemiluminometric technology using kits (ADVIA Centaur) supplied by (Bayer Health Care Diagnostics, USA). The test was performed according to the manufacturer’s instructions.

Follow up of all patients was carried out until 20 weeks of pregnancy to detect the patients who would miscarry and those who would proceed into the second trimester. Then, comparison was done between the two study groups, miscarried and continued groups, for ultrasound finding data, progesterone level and CA-125 level.

### Statistical analysis

Data were statistically described in terms of range, mean ± SD, median, frequencies (number of cases) and percentages when appropriate. Comparison of numerical variables between the study groups was done using one-way ANOVA test with posthoc multiple 2-group comparisons. For comparing categorical data, Chi square (χ2) test was performed. Exact test was used instead when the expected frequency is less than 5. Accuracy was represented using the terms of sensitivity, and specificity. Receiver operator characteristic (ROC) analysis was used to determine the optimum cut off value for the studied diagnostic markers. P values less than 0.05 was considered statistically significant. All statistical calculations were done using computer programs Microsoft Excel 2007 (Microsoft Corporation, USA) and SPSS (SPSS Inc., USA) version 15 for Microsoft Windows.

## Results

A total of 100 pregnant patients with vaginal bleeding between 7 and 13 weeks’ gestation in which a singleton embryo with cardiac activity was initially documented completed the study. Twenty cases ended by miscarriage (20%, group 1) and 80 cases (80%, group 2) continued till 20 weeks of gestation.

No statistically significant differences were found between both groups as regards maternal age, parity, the number of previous miscarriages, and CRL. The mean GSD was significantly lower in the group that miscarried compared to the group that continued (P=0.023, Table 1). The mean FHR was 156.9 ± 20 bpm for the continued group and 122 ± 9 for the aborted group, which showed a statistically significant difference (P<0.001).

On comparison between study cases presented by sub chronic hematoma in relation to study parameters, CA-125 level was significantly higher in the group that showed hematoma as compared with the group without hematoma (52.857 ± 29.219 vs. 23.501 ± 13.295, P<0.001). Also, serum progesterone level was significantly lower in the group that showed hematoma as compared with the group without hematoma (14.67 ± 7.09 vs. 23.507 ± 9.39, P=0.017).

The level of serum CA-125 for the threatened miscarriage (miscarried) group was 54.28 ± 11.4 IU/ml; while for the threatened miscarriage (continued) group it was 18.81 ± 8.02 IU/ ml. The difference was statistically significant (P<0.001). The level of serum progesterone for the threatened miscarriage (miscarried) group was 8.7 ± 1.85 ng/ml; while for the threatened miscarriage (continued) group it was 26.3 ± 7.2 ng/ml, which showed a statistically significant difference (P<0.001, Table 1).

Using a ROC curve for CA-125 in predicting the outcome of pregnancy in threatened miscarriage cases, the cut-off limit of 31.2 IU/ml of CA-125 level achieved sensitivity of 96.2% and specificity of 100%. CA-125 level above 31.2 IU/ml predicted occurrence of miscarriage with an overall accuracy of 99.4%.

Using a ROC curve for progesterone level in predicting the outcome of pregnancy in threatened miscarriage cases, the cut-off limit of 11.5 ng/ml of progesterone level achieved sensitivity of 97.5 % and specificity of 100%. A progesterone level of <11.5 ng/ml predicted the occurrence of miscarriage with an overall accuracy of 99.8%.

CA-125 level showed a strong significant negative correlation with progesterone level (r=-0.716, P<0.001), and a significant negative correlation with the FHR (r=-0.414, P<0.001). Serum progesterone level showed a correlation with GSD (r=0.521, P<0.001), CRL (r= 0.407, P<0.001) and FHR (r= 0.363, P<0.001, Table 2).

**Table 1:** Ultrasonographic and biochemical markers in the miscarried and continued pregnancy groups


	Group 1 (miscarried)	Group 2 (continued)	P value

Gestational age (weeks)	8.8	9.2	0.304
GSD (mm)	30.03	37.7	0.023*
CRL (mm)	24	28.5	0.317
FHR (bpm)	122	156	<0.001*
Presence of SCH	5	2	0.002*
Serum CA-125 level (IU/ml)	54.280	18.81	<0.001*
Serum progesterone level (ng/ml)	8.716	26.317	<0.001*


*; Significant difference (P<0.05), GSD; Gestational sac diameter, CRL; Crown-rump length, FHR; Fetal heart rate, SCH; Subchorionic hematoma and CA-125; Cancer antigen-125.

**Table 2:** Correlation between CA-125 and progesterone levels to the other study parameters


	CA-125 level		Progesterone level	
	R	P value	R	P value

Age	-0.029	0.774	0.090	0.374
Gestational age	0.072	0.475	0.382	<0.001*
CRL	0.035	0.739	0.407	<0.001*
GSD	-0.042	0.680	0.521	<0.001*
FHR	-0.414	<0.001*	0.363	<0.001*


*; Significant difference (P<0.05), CRL; Crown-rump length, GSD, Gestational sac diameter, FHR; Fetal heart rate and CA-125; Cancer antigen-125.

## Discussion

The present study aimed to evaluate the prognostic value of serum progesterone level and serum CA-125 level at the time of initial presentation with pregnancy outcome in patients with first trimester threatened miscarriage.

As regards GSD, the GSD of the group that continued pregnancy was significantly higher than that of the group that miscarried. These results are also in agreement with the study by Falco et al. (6) who evaluated the outcome and prognostic criteria of pregnancies with first-trimester bleeding and a gestational sac ≤16 mm. They found that of 50 patients, 32 (64%) underwent miscarriage. The size of GSD a high level of statistical significance.

However, these results are not in agreement with the study by Oh et al. (7) who found that the mean diameter of the gestational sac at 28-42 days from the last menstrual period among normal pregnancies did not differ significantly from that in those that subsequently miscarried (2.6 vs. 2.7 mm). This difference can be attributed to the difference in the range of gestational age at which ultrasound was done, 4-6 weeks in their study and 7-13 weeks in our study.

In this study, the CRL was not significantly different between the group that continued pregnancy and the group ended by miscarriage (P=0.06), which was inconsistent with Reljic (8) who studied 310 singleton pregnancies with live fetuses, presenting with threatened miscarriage before 13 weeks of gestation. He reported that in fetuses with CRL<18 mm, there was a significant positive association between deficit in the CRL for gestation and the incidence of subsequent spontaneous miscarriage. The smaller number of women in our study may explain this difference.

In this study, there was a significant difference between women who miscarried and women who continued regarding the presence of sub-chorionic hematoma (SCH) (P=0.002). These results are in agreement with many studies that showed that SCH was associated with high incidence of 1st trimester miscarriage (9, 10). However, our results are not in quite agreement with Pearlstone and Baxi’s findings (9). They reviewed the English literature on SCH. Fourteen studies were reviewed. The incidence of SCH varied greatly among studies from 4 to 48 per cent. They concluded that small SCH tend to be more common in the first trimester and appear to pose no added risk to the ongoing pregnancy but this could be challenged by how small the hematoma needed to be so that to have no adverse effects. Also we didn’t correlate the size and site of the hematoma with the outcome, which is a limitation of our study.

In this study, the FHR was significantly different between the two groups (the miscarried and the continued groups). Our results are in agreement with Doubilet and Benson’s findings (11). However, when the embryonic heart rate is within the normal range for gestation, the outcome remains uncertain, as in another study done by Tannirandorn et al. (12).

The concentrations of CA-125 in the pregnant women who subsequently miscarried were higher than those who did not, thus suggesting that the serumCA-125 levels are not so important in maintaining successful pregnancy (13). CA-125 might have a role in the preparation of the endometrium for successful implantation (14). More trophoblastic damage is associated with higher levels of CA125 and lactate dehydrogenase (LDH) (15). CA-125 can be used as a prognostic factor to the outcome of pregnancy as it might be related to the extent of trophoblastic destruction.

In the present study, serum CA-125 levels showed a significant difference between the group of women that continued and the group of women that miscarried (P<0.001). These results are consistent with other studies (14-16). There was a highly significant increase in serum CA-125 level in women who miscarried. They stated that serum CA-125 level might be developed as a cheap, sensitive and specific predictor of outcome in cases of threatened miscarriage, whereas Mahdi (16) found that there was no statistically significant difference in CA-125 level of patients who miscarried compared with those women that continued pregnancies in spite of its higher level. Their study showed that serum CA-125 level are not predictive of spontaneous miscarriage in the first trimester and failed to discriminate among threatened miscarriages and normal pregnancies.

Several cut-off values were suggested in other studies in order to predict pregnancy outcome in early viable pregnancies complicated by vaginal bleeding or to discriminate between viable and non-viable gestations at the time of vaginal bleeding. In this study, a cut-off limit of 31.2 IU/ml of CA-125 level was suggested, with a sensitivity of 96.2% and specificity of 100%. Fiegler et al. (17) used a cut-off value of 66.5 IU/ml with a sensitivity of 55%. Schmidt et al. (18) used 65 IU/ml as a cut-off value and reported a sensitivity of 50% for this level. Azougi et al. (19) used a 125 IU/ml as a cut-off value and reported a 100% sensitivity and specificity.

The present study evaluated the possible role of serum progesterone measurement in the prognosis of first trimester miscarriage. According to the statistical analysis, there was a significant difference between the group of women that continued and the group of women that miscarried (P<0.001). This was in accordance with the study of Edwar et al. (20) who studied 78 pregnant women presented by vaginal bleeding, 44 continued till 13th week of pregnancy and 34 ended with spontaneous miscarriage. Serum progesterone level was 5.7 ± 10.9 in continuing pregnancy and 6.7 ± 4.8 in spontaneous abortion. The difference in progesterone level was highly significant.

## Conclusion

The use of certain maternal serum markers (CA- 125 and progesterone) in the first trimester represent non invasive, early and fast methods that can be considered as a good predictor for the outcome of pregnancy in cases with threatened abortion, Larger clinical trials are still needed to support this recommendation.
